# Comparison of Resting-State EEG and Synchronization Between Young Adults with Down Syndrome and Controls in Bipolar Montage

**DOI:** 10.3390/brainsci16030328

**Published:** 2026-03-19

**Authors:** Jesús Pastor, Lorena Vega-Zelaya, Diego Real de Asúa

**Affiliations:** 1 Clinical Neurophysiology and Instituto de Investigación Biomédica, Hospital Universitario de La Princesa, C/Diego de León 62, 28006 Madrid, Spain; lorenacarolina.vega@salud.madrid.org; 2 Servicio de Medicina Interna and Instituto de Investigación Biomédica, Hospital Universitario de La Princesa, C/Diego de León 62, 28006 Madrid, Spain; diego.realdeasua@salud.madrid.org

**Keywords:** alpha band, coherence, cross-correlation, fast Fourier transform, Pearson’s correlation coefficient, posterior dominant rhythm, power spectrum, Shannon’s spectral entropy

## Abstract

The qEEG findings of subjects with Down syndrome (DS) have not been described in the context of bipolar montage. Resting-state EEG (rsEEG) with a bipolar montage was performed in 22 young adults (26.0 ± 1.2 years) with DS but without psychiatric or neurological pathology and matched control subjects of the same sex and age, and the results were conventionally and numerically analyzed. Channels were grouped into frontal, parieto-occipital, and temporal lobes. For every channel, the power spectrum was calculated and used to compute the area for the delta, theta, alpha and beta bands and was log-transformed. Shannon’s spectral entropy (SSE) and coherence by bands were computed. Finally, we also calculated the peak frequency distribution of the alpha band. qEEG revealed alterations in the rsEEG that were not detected visually. Subjects with DS showed a significant generalized increase in the power of the delta and theta bands, along with a decrease in the power of the alpha band in the posterior half of the scalp. This alpha activity also exhibited features corresponding to older euploid subjects, showing interhemispheric asynchrony in one-third of the individuals. The beta band power was significantly increased in the frontal lobes and adjacent regions, such as the parietal and mid-temporal regions. Individuals with DS showed a generalized decrease in parieto-occipital synchronization associated with intelligence quotient. Left temporal synchronization was also lower. The synchronization of specific channel pairs was greater in subjects with DS in the frontal lobe and much lower in the occipital and temporal regions. These results indicate that alterations in band structure and synchronization in subjects with DS are highly specific and can aid in the clinical evaluation of these individuals.

## 1. Introduction

Down syndrome (DS) is a genetic condition characterized by an extra copy of chromosome 21; its prevalence (8% of congenital anomalies) is increasing, and it occurs in approximately one in every 700 live births [1]. The life expectancy of patients has tripled (from 19 to 66 years old) in the past 30 years [2]. In DS, cognitive impairments manifest as reduced memory and executive functions [3,4]. Acquisition of language and writing are delayed, and mental age in adults does not exceed 8 years, with an intelligence quotient (IQ) usually under 70. These life-long cognitive dysfunctions result in important negative consequences for people with DS, their families, and society. Research is ongoing to fully elucidate the complex underlying causes of these cognitive deficits, which involve anomalies in brain connections and neuronal circuits [5].

EEG is the fastest, least expensive and most portable method for noninvasively measuring brain activity. The temporal resolution is fitted to nervous system dynamics, and this approach can be easily implemented in the hospital environment; the results of such tests can help researchers identify critical aspects of brain function in individuals with DS [6,7,8]. Analysis of particular EEG patterns can aid in the identification of potential biomarkers for cognitive deficiencies or risk factors for the development of neurodegenerative illnesses such as Alzheimer’s disease, one of the most frequent comorbidities in adults with DS [9]. Identifying these indicators may aid in early diagnosis and treatment, possibly improving outcomes for individuals with DS.

Resting-state EEG (rsEEG) is a powerful method for assessing the structure of waveforms and synchronization across the scalp; rsEEG results depict the baseline homeostatic system under physiological conditions. Consequently, rsEEG patterns serve as an endophenotype of brain malfunctions and neural network alterations [10,11]. The waveform structure can be defined as the arrangement of and relationships between the parts or elements of a complex system. We can apply this concept to the EEG as the arrangement and relationships of the different bands (delta, theta, alpha, and beta) and synchronization across the whole scalp [12]. Although several studies have addressed the structure of brain oscillations in DS subjects (see [8] for a review), all of them have been performed with unipolar recordings. However, a bipolar montage is more frequently used in clinical practice. Nevertheless, the way in which we obtain the EEG is fundamental for the quantitative values of the power spectrum (PS) and synchronization must be considered [12,13]. Therefore, the PS and synchronization values differ between bipolar and unipolar recordings. Consequently, it is necessary to describe the features of subjects with DS in bipolar montages to make the results useful for guiding future clinical studies.

Research on EEG in individuals with DS has consistently shown elevated activity in the delta and theta frequency bands, although anomalies in the alpha and beta frequency bands have also been described [8]. In this sense, several studies have indicated that people with DS exhibit lower amplitude and power of alpha activity in the posterior brain regions. Conversely, beta activity is inconsistent among DS patients, with some studies reporting increased amplitude and others suggesting reduced beta activity [8]. Age-dependent alterations in the posterior dominant rhythm (PDR) have also been described.

Studies concerning synchronization are scarce and inconclusive, although the topology of the functional connectivity of DS patients with disruptions in the whole brain in the alpha and theta bands has been described, together with changes in coherence [14,15] and interhemispheric functional coupling [16]. As indicated above, these studies have also been carried out in a reference setup.

Our objectives are the following:

1. Compare the results of the conventional visual rsEEG analysis and the quantitative analysis (qEEG), according to the usual diagnostic categories (i.e., physiological recording, slowing (mild or moderate) and encephalopathy).
2. Evaluate the hypothesis (H_0_) that there are no differences between men and women with DS in the distribution of scalp bands.
3. To evaluate in detail the peak frequency and interhemispheric synchronization characteristics of posterior alpha activity in subjects with DS.
4. Evaluate in detail the properties of the beta band in scalp (i.e., topographic distribution and magnitude relative to other bands).
5. Describe the scalp properties of synchronization for Pearson correlation coefficient and coherence by bands metrics.
6. Evaluate the hypothesis of an asymmetric transfer of information between symmetric interhemispheric regions.
7. If rsEEG can be considered as an endophenotype of physiological and pathological brain function, it would be expected that there would be some relationship between the overall function of cognitive processes, determined by IQ, and the structure of EEG bands or synchronization.

All targets have been analyzed in a double-banana bipolar montage, which is widely used in daily clinical practice, in a group of young patients with DS and matched control subjects of the same age and sex.

This is a descriptive work, preliminary to the future development of clinical applications.

The abbreviations used are listed at the end of the manuscript.

## 2. Materials and Methods

### 2.1. Participants

In this study, we analyzed 20 min rsEEG recordings obtained from 14 men (25.6 ± 1.5 years old) and 8 women (30.4 ± 2.8 years old) diagnosed with DS on the basis of genetic studies. According to the literature, this age range corresponds to young adults [8]. The exclusion criteria included previous neurological or psychiatric disturbances and treatment with psychoactive drugs. The IQ of this group was evaluated with the Kaufmann Brief Intelligence Test, version 2 (KBIT-2), and the mean score was 46.8 ± 2.81, ranging from 33 to 74.

The clinical procedure was nested in a clinical trial approved by the institutional review board of the Parc de Salut MAR (protocol code AEF0217-102, EudraCT number 2022-000923-19). Written informed consent was obtained from the parents or legal guardians of all DS subjects. Participants also provided written assent or consent if they showed the capacity to provide it. Nine of these subjects were treated with low doses of levothyroxine, and another partially different subgroup of nine individuals were diagnosed with obstructive sleep apnoea, three of whom required treatment with positive pressure.

Each of these subjects was matched by age and sex with a control participant. These subjects in the control group (CG) did not present any neurological or psychiatric pathology, and no pharmacological treatment affecting the brain was followed.

### 2.2. EEG Recording and Analysis

Eye-closed rsEEG records were obtained while the subjects were seated comfortably in a sound- and light-attenuated room using a 32-channel digital EEG system (EEG32U, NeuroWorks. XLTEK^®^, Oakville, ON, Canada) with a 19-electrode cap placed according to a 10–20 international system. In addition, the differential derivation I of Einthoven for ECG was positioned. Great care was taken to calm the DS subjects to obtain the best possible recording. To this end, parents usually accompanied the subject during the study and were sometimes asked to keep their fingertips on the subject’s eyebrows to minimize blinking. Special care was taken to minimize the possible artefact generated by this contact, which was only maintained in those cases where it was verified that the contact did not modify the record (except for the minimization of the artefact). Recordings were performed at a 512 Hz sampling rate, with a filter bandwidth of 0.5 to 70 Hz and a notch filter of 50 Hz. Electrode impedances were usually less than 15 kΩ.

**Figure A1:**
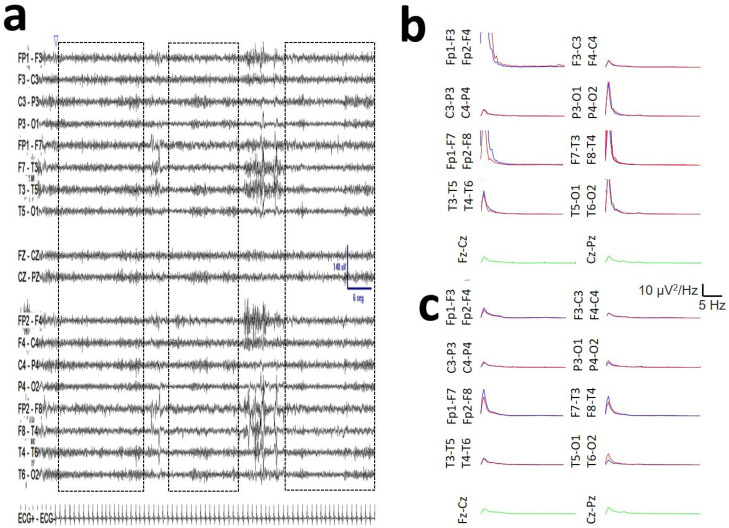
Exclusion of different artefacts by visual selection in a DS individual. (**a**) Raw recording with artefact and non-artefact periods (dashed lines). (**b**) Average spectra from the whole recording and (**c**) average spectra from the non-artefact period selected from dashed lines. Red = right hemisphere, blue = left hemisphere, green = midline.

The numerical method used for qEEG has been previously described in detail elsewhere [12,17,18]. Briefly, different lengths of raw records were exported from the EEG device to the ASCII file. The minimum recording time analyzed was 120 s. Artefacts of electro-oculography, electromyography, electrode contact or movement were visually identified and, consequently, were excluded (Appendix A, Figure A1) by the export of several artefact-free chunks, which were later combined for analysis. These waveforms are highly specific and can be identified easily by any experienced clinical neurophysiologists. The raw recordings were digitized, exported and analyzed at 512 Hz. Exported files were digitally filtered by a sixth-order Butterworth digital filter between 0.5 and 30 Hz.

A differential EEG double-banana montage was set up. The topographic placement of channels was defined on the scalp as the midpoint between the electrode pairs defining the channel, e.g., the Fp1–F3 channel was placed at the midpoint of the geodesic between the Fp1 and F3 electrodes. In a group of 10 subjects with DS, the channels were not recombined in a bipolar montage but were analyzed using a referential average montage (AVG).

All the recordings were divided into one-second moving windows with 10% overlap. This method has been used in previous work [6,12,13,17,18], and no stationarity problems have been observed. Robustness of the method regarding different lengths of windowing (1, 3 and 5 s), overlap (10, 20 and 40%) and sample frequency (128 and 512 Hz) has been previously confirmed (please see the paragraph Robustness of the method in [6]). This windowing allows a spectral resolution of 0.5 Hz, which is suitable for clinical practice and a sufficiently large number of elements (a minimum of 132) for the stationary distribution to have Gaussian characteristics. The total length used during the fast Fourier transform (FFT) is directly related to the frequency precision in the PS. Overlap was used to minimize the effect produced by windowing. These features give rise to a maximum frequency sensitivity of 0.5 Hz.

For each window and frequency (*k*), we computed the discrete FFT of the voltage obtained from every channel to obtain the PS (in µV^2^/Hz). We also computed Shannon’s spectral entropy (SSE) for every m-channel according to the following expression:

(1) $${SSE}_{k}^{m}=-\sum_{k=0}^{F}p_{k}\log_{2}p_{k}$$

where *F* is the maximum frequency computed and *p_k_* is the probability density of the PS (*S^m^*), obtained from the following expression:

(2) $$p_{k}=\frac{S_{k}^{m}}{\sum_{k=0}^{F}S_{k}^{m}∆k}$$

Before SSE computation, PS were normalized to avoid the entropy values were biased by the different distribution amplitudes.

We used the classical segmentation of EEG bands (in Hz): delta (δ): 0.5–4, theta (θ): 4–8, alpha (α): 8–13 and beta (β): 13–30. The different EEG bands are rooted in different neural systems; therefore, the synchronization of different bands can offer specific information. Linear synchronization in the time domain was assessed by Pearson’s correlation coefficient. A very useful method to assess specific band synchronization in the frequency domain for channels i and j is coherence (coh), computed according to the next expression:

(3) $$coh\left(\omega \right)=\frac{{\left|{\langle S_{ij}\left(\omega \right)\rangle }_{k}\right|}^{2}}{{\langle S_{ii}\left(\omega \right)\rangle }_{k}{\langle S_{jj}\left(\omega \right)\rangle }_{k}}$$

Window length and overlap percentage were the same as used for spectral analysis.

These methods were used to compute the average synchronization at a defined anatomical region. Cross-correlation (CC) is a metric derived from Pearson’s correlation, but it includes a time-dependent variable that Pearson’s correlation lacks. This allows for an estimation of the directionality indicated by the sign and magnitude of the maximum lag and has been used as an estimate of the possible directionality and average value of interhemispheric synchronization between symmetrical pairs of frontal, parietal, occipital, and temporal channels [13].

The mean values of all the windows for Pearson’s correlation and coh were computed, and the mean correlation and coherence matrices were obtained. The mean values of synchronization for the hemispheres and lobes were computed as the average of all pairs ($N_{pairs}$) of channels ($N_{ch}$), according to the expression $N_{pairs}=\frac{N_{ch}\left(N_{ch}-1\right)}{2};N_{ch}=3\;for\;lobes\;and\;N_{ch}=9$ for the hemispheres.

The spectral and synchronization variables were topographically grouped into hemispheres and lobes. In the case of the left hemisphere (shown as an example), we grouped as follows for the next topographical regions:

(4) $$\mathrm{Frontal}\;\mathrm{lobe}\;F=\left\{\frac{\left({Fp}_{1}-F_{3})+(F_{3}-C_{3})+({Fp}_{1}-F_{7}\right)}{3}\right\}$$

(5) $$\mathrm{Parieto}-\mathrm{occipital}\;\mathrm{lobe}\;PO=\left\{\frac{(C_{3}-P_{3})+(P_{3}-O_{1})+\left(T_{5}-O_{1}\right)}{3}\right\}$$

(6) $$\mathrm{Temporal}\;\mathrm{lobe}\;T=\left\{\frac{\left(F_{7}-T_{3}\right)+\left(T_{3}-T_{5}\right)+\left(T_{5}-O_{1}\right)}{3}\right\}.$$

For the whole left hemisphere, we used the expression

(7) $$H=\left\{\frac{\left({Fp}_{1}-F_{3}\right)+\left(F_{3}-C_{3}\right)+\left({Fp}_{1}-F_{7}\right)+\left(C_{3}-P_{3}\right)+\left(P_{3}-O_{1}\right)+\left(T_{5}-O_{1}\right)+\left(F_{7}-T_{3}\right)+\left(T_{3}-T_{5}\right)+\left(T_{5}-O_{1}\right)}{9}\right\}.$$

Channels from the right hemisphere were grouped accordingly.

In the AVG setup, the average values for each lobe were grouped using the same electrodes (in this case, channels) as those used in the bipolar montage. This allowed for a comparison of the average logPS value in both types of montages. As an example of PS for the frontal lobe in the AVG montage,

(8) $$F=\left\{\frac{{Fp}_{1}+F_{3}+C_{3}+F_{7}}{4}\right\}$$

**Figure A2:**
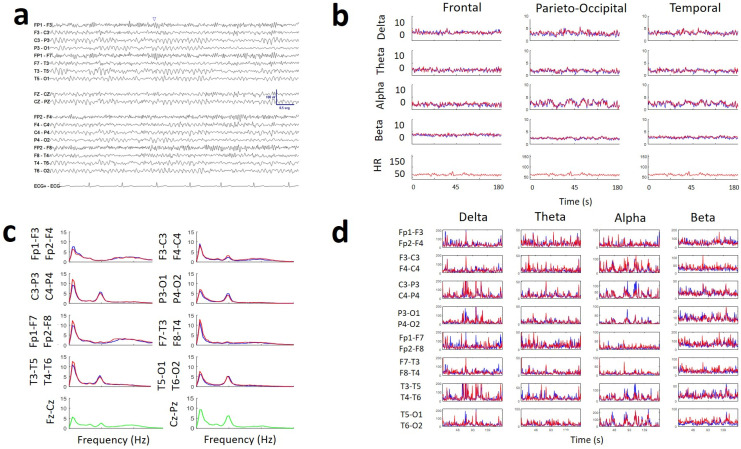
Grouping of channels by lobes. The same effects are observed at lobes, spectra and individual channels. (**a**) Raw recording in a 31-year-old subject with DS. (**b**) Dynamics of the EEG bands grouped by lobes for a 180 s recording. (**c**) Average spectra for each channel over the complete recording. (**d**) Dynamics of the bands by channel. Red = right; blue = left; green = midline.

The spatial averaging can reduce the topographic resolution. However, these lobe-level effects are not driven by a single derivation. In fact, the same features are observed for individual channels and average lobes (see Appendix A, Figure A2).

We addressed the interhemispheric synchronization between paired lobes. For this, we selected F (F3–C3/F4–C4), parietal (P; C3–P3/C4–P4), temporal (T; T3–T5/T4–T6) and occipital (O) paired channels (P3–O1/P4–O2) to compute the cross-correlation (CC) and the maximum lag [13].

Posterior dominant rhythm (PDR) is typically defined as the peak frequency of the power spectrum (pPS) for the 7.5–13 Hz band at the P3–O1 and P4–O2 channels that is blocked during eye opening. In addition, we broadly assessed this feature to evaluate the stability of pPS in the alpha band in adjacent parietal regions (C3–P3 and C4–P4) and the midline (Cz–Pz).

Pseudocode, a step-by-step description of the algorithm using a mix of programming language conventions and informal notation, is placed at https://osf.io/2ndr7/files/osfstorage, accessed on 15 March 2026.

The test–retest reliability of this method has been addressed in a previous publication for logPS, coherence and SSE, obtaining coefficients of stability higher than 0.900 [12].

Numerical analysis of the EEG recordings was performed with custom-made MATLAB^®^ software, 2021 release (MathWorks, Natick, MA, USA).

### 2.3. Statistics

Evidently, the absolute values of the PS are quite different from patient to patient; therefore, it is not useful to compare the raw values. Therefore, we used a log transform [12,18,19] for the PS measures (logPS). The synchronization measures and SSE were compared in terms of their raw values.

Statistical comparisons between the DS and CG groups were performed by paired Student’s *t* test for normally distributed data. Normality was evaluated by the Shapiro-Wilk test, and equal variance was evaluated by the Brown–Forsythe test. Comparisons between men and women in the DS group were made via two-tailed Student’s *t* test for data following a Gaussian distribution and via the Mann–Whitney rank sum test when the date were non-normally distributed. Comparisons among three groups were made via Kruskal-Wallis one-way analysis of variance on ranks and Dunn’s post hoc test for paired averages. SigmaPlot^®^ v16 software (Grafiti LLC, Palo Alto, CA, USA) and MATLAB^®^ were used for statistical analysis. Multivariate comparisons by means of one-way RM ANOVA on ranks were performed with Bonferroni correction.

To evaluate the PDR, measured at P3–O1/P4–O2, we identified the peak frequency in the PS (pPS_channel_) in the alpha band, between 8 and 13 Hz, for five different bipolar channels (C3–P3, Cz–Pz, C4–P4, P3–O1 and P4–O2). We assessed the average alpha (${alpha}_{p}$) at parietal regions using this expression:

(9) $${alpha}_{p}=\frac{{pPS}_{C3-P3}+{pPS}_{Cz-Pz}+{pPS}_{C4-P4}}{3}$$

The average occipital alpha (alpha_o_) was calculated according to

(10) $${alpha}_{o}=\frac{{pPS}_{P3-O1}+{pPS}_{P4-O2}}{2}$$

The interhemispheric difference (ihDif) was assessed as follows:

(11) $$ihDif={pPS}_{P3-O1}-{pPS}_{P4-O2}$$

We also computed the difference between the left parietal midline channel and the parieto-occipital channel mlPO_left/right_ as follows:

(12) $${mlPO}_{left}={pPS}_{Cz-Pz}-{pPS}_{P3-O1}$$

(13) $${mlPO}_{right}={pPS}_{Cz-Pz}-{pPS}_{P4-O2}$$

Finally, we computed the standard deviation (SD) for all five channels.

The best fit to the PDR data from DS subjects (${PDR}_{DS}$) to the normative equation (PDR(age)) in control subjects [12] was achieved as follows. Initially, we considered the group of DS subjects for ${PDR}_{DS}$, defined as ${PDR}_{DS}=\left\{\left({age}_{i},{freq}_{i}\right);i=1,2,\ldots 22\right\}.$ For every point, we computed the squared of the vertical (frequency dimension) difference with the function and then summed it for all the groups (D) according to the following expression:

(14) $$D=\sum_{i=1}^{22}{\left(PDR\left({age}_{i}\right)-{freq}_{i}\right)}^{2}$$

Finally, we minimized this magnitude numerically, obtaining an age (in fact, the mass centre for the whole group) that minimized this value. We then used this value to plot ${PDR}_{DS}$ at this point.

The average PS was determined by normalization of all the PS in every subject to the maximum amplitude of the PS at Cz–Pz. We selected this channel because it is located away from the eyes, minimizing electrooculogram artefacts, and because its midline location is away from the insertion of the temporal and occipital muscles, minimizing muscle artefacts.

Quadratic nonlinear regression was performed by means of the least-squared method, and correlation coefficient was used to study the dependence between variables. Statistical significance was evaluated by means of a contrast hypothesis against the null hypothesis using the formula

(15) $$t=\frac{r\sqrt{n-2}}{\sqrt{1-r^{2}}}$$

This describes a one-tailed *t*-Student distribution with *n* − 2 degrees of freedom [12].

**Figure A3:**
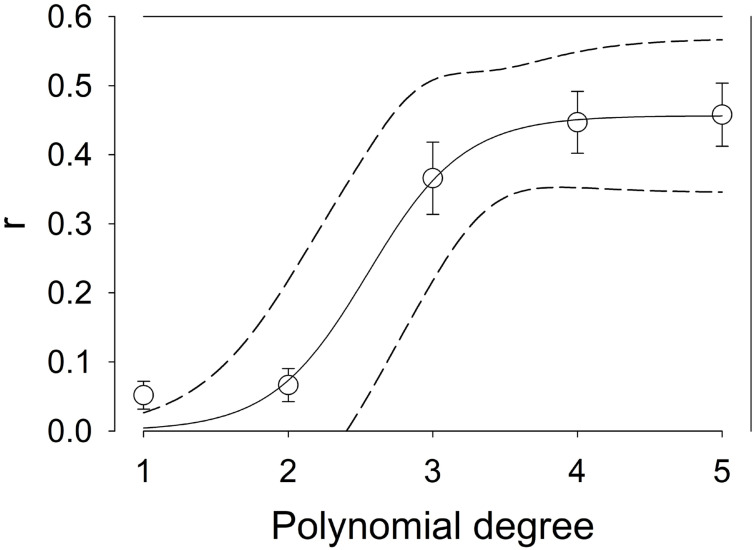
Relationship between polynomial degree and correlation coefficient (r) for functions obtained from both PO lobes (n = 10). The sigmodal function (continuous line) is $r\left(degree\right)=\frac{0.4565}{1+e^{-\frac{degree-2.5499}{0.3327}}};r=0.9929$; dashed line = 95% confident band.

Fitting to polynomial functions carries the risk of overfitting, which can occur by increasing the number of polynomial terms. To minimize this possibility, we followed these steps:

1. We fit the polynomial function regression between the first (linear function) and fourth polynomial degree (x^4^) and find the values of r. We used data from PO lobes and all the metrics tested (n = 10).
2. We represent all these averaged data in a graph with the degrees of the polynomials on the x-axis and the values of r on the y-axis fitted to a sigmoidal function (Appendix A, Figure A3).
3. From that figure, it is evident that the minimum polynomial degree explaining more than 75% of r is 3, because a degree of 2 explains below 15%, an obviously underfitted result.

Descriptive statistics are presented as the mean ± SEM (for Gaussian distributions) or median (Med) and the interpercentile range IP25–75 (percentile 25, percentile 75) for non-Gaussian distributions.

The significance level was set at *p* = 0.05.

## 3. Results

Recordings were visually examined by expert clinical neurophysiologists (JP and LV-Z, who have more than 10 years of experience) in a conventional manner before being subjected to numerical analysis, blinded to the condition of the subject. They were evaluated according to conventional criteria [20,21], basically addressing the presence of an anteroposterior gradient (present/absent) and RDP (alpha/theta/absent), including reactivity (present/absent) and excess delta/theta rhythm (no/anterior/generalized). With respect to these variables, the results were classified as physiological, mild slowing, moderate slowing and encephalopathy. No epileptic waveforms were observed in any recording. The features of each of these EEG-defined states are detailed in the Appendix A table (see Appendix A, Table A1).

**Figure 1:**
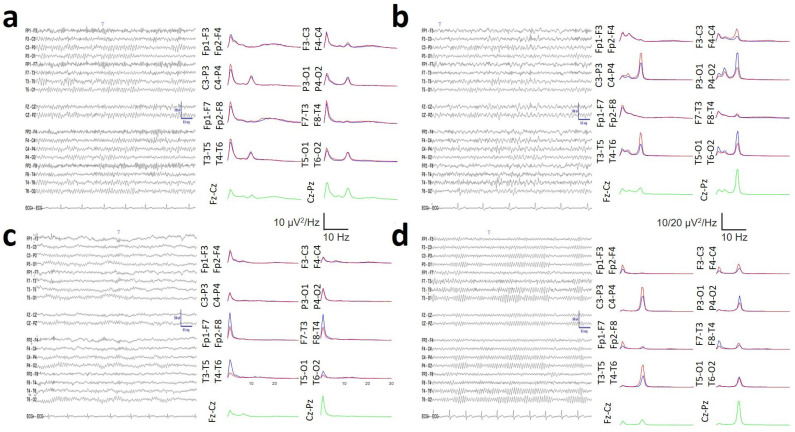
Visual inspection of the rsEEG and associated PS. (**a**) Raw trace of a DS classified visually as physiological; (**b**) mild slowing; (**c**) encephalopathy and (**d**) control subject corresponding to (**a**). Red = right hemisphere, blue = left hemisphere, green = midline.

**Figure 2:**
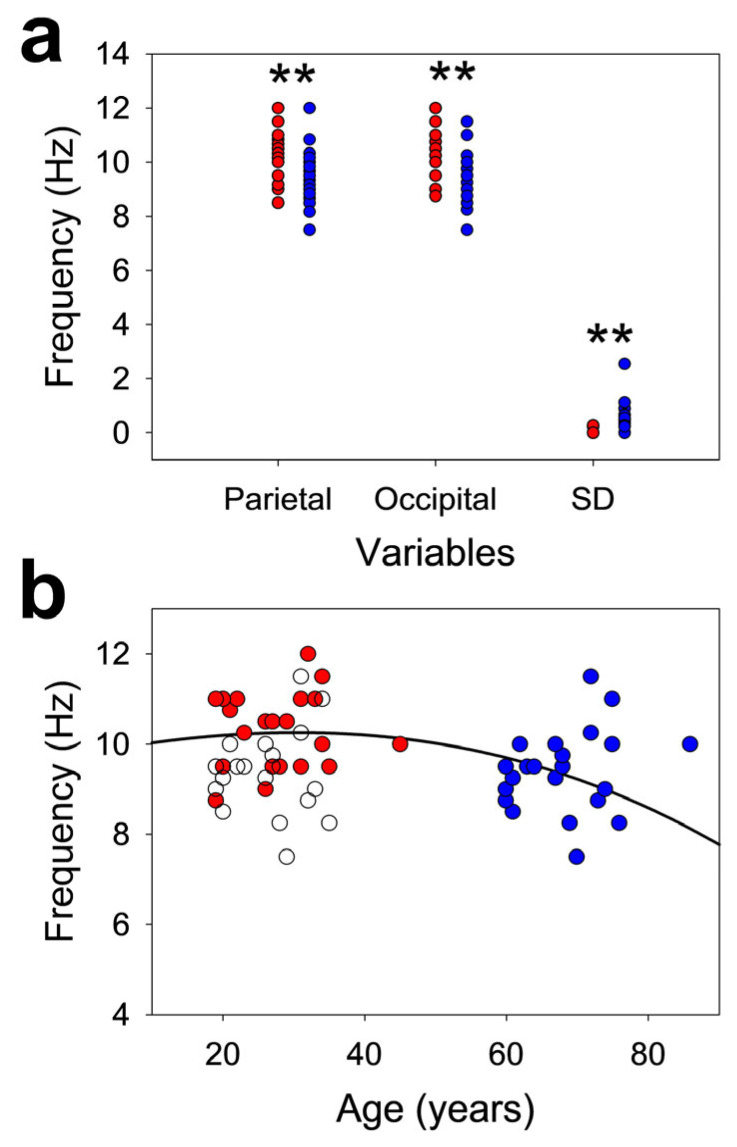
Properties of the alpha band. (**a**) Scatter plot for the pPS values in the parietal and occipital regions and SDs, ** *p* < 0.01; (**b**) graph dispersion for the CG (red dots) and for the DS group (empty dots). The blue dots represent the same DS group displaced to a better fit to the normative function for the PDR. The solid line corresponds to the normative function for PDR in control subjects [12].

No changes in PS were observed in DS subjects with treatment with levothyroxine or diagnosis of sleep apnoea (see Appendix A, Table A2). In general, the rsEEG recordings from DS subjects revealed a physiologic or nearly physiologic pattern in 10/22 subjects (48%), with the presence of an antero-posterior gradient and a reactive PDR and no visual excess of delta/theta activity and no asymmetries (Figure 1a). In 5/22 subjects (24%), mild slowing was indicated by the presence of theta activity in the anterior region and/or a PDR lower than 8 Hz (Figure 2b). When the theta activity spread across the scalp, moderate slowing was diagnosed in 4/22 of the subjects (19%). Only in two patients (9%) was a diagnosis of mild encephalopathy attained (Figure 1c). 

No significant changes in PDR power were observed with age in either the DS subjects or the control group. Linear regression was performed between power and age, and no significant differences were found for these functions $\left({PRD}_{CG}=10.24+0.0034\;years,\;p=0.906\;and\;{PRD}_{DS}=8.60+0.0297\;years,\;p=0.3304\right)$. No differences between functions were observed. Therefore, there is no evidence of changes in this parameter within this age range.

However, numerical analysis of all the DS records revealed several anomalies in the spectral pattern that were not easily identifiable by visual analysis. In this way, Figure 1a shows an example of an rsEEG recording from a DS subject classified as physiologic. The average PS for every channel is shown to the right of every raw record. In Figure 1b, we show mild slowing; in Figure 1c, we present an example of encephalopathy. Finally, Figure 1d shows the control subject from Figure 1a. Although the results of the de visu analysis were quite similar between the physiological DS and the CG subject records, the average spectra clearly indicate a generalized increase in the delta band power and a marked increase in the beta band power in the frontal lobes. These features clearly differ from the spectra obtained from the control subject (Figure 1d).

Therefore, from results similar to this example, it is evident that a nearly physiological recording in de visu analysis in DS subjects can have, in fact, several alterations in its numerical analysis.

### 3.1. Comparison by Sex

We addressed the similitude of the rsEEG for both sexes. To do that, we compared the average values of logPS for all the bands and SSE and for the left and right lobes (Table 1).

Out of a total of 30 possibilities, only two paired variables were (minimally) statistically significant. Therefore, we can conclude that no significant differences in the band structures between males and females were observed. Therefore, for the remainder of the study, both sexes were considered together for comparison with the control group.

### 3.2. Posterior Dominant Rhythm and Alpha Band

We assessed the symmetry in the alpha band and the PDR and the variability in the occipital and adjacent parietal regions.

All the pPS values for the alpha band in DS subjects were lower than those in the CG (Figure 2a). The average values for the CG and DS groups were alpha_p,CG_ = 10.4 ± 0.2 Hz and alpha_p,DS_ = 9.4 ± 0.2 Hz, respectively (*p* = 0.0038, paired Student’s test), and for the occipital areas, alpha_o,CG_ = 10.3 ± 0.2 Hz and alpha_o,DS_ = 9.5 ± 0.2 Hz, respectively (*p* = 0.0012, paired Student’s *t* test). In addition to having a lower average PDR in DS subjects, the dispersion of pPS was higher, with a value and SD of 0.47 ± 0.12 Hz in the DS group; notably, these values were approximately three times higher in the control group (0.16 ± 0.03 Hz; *p* = 0.002, paired Student’s *t* test).

We fitted the distribution of the PDR_DS_ to the best age interval [12], obtaining an average mass centre of 68 years instead of 27.3 ± 1.4 years for the original ages of the group. Therefore, the DS group was more similar to subjects approximately 68 years old than to young adults.

Moreover, we addressed several other properties, as shown in Table 2. The number of asynchronous PDRs for control subjects was approximately one-third (8 subjects for DS and 3 for CG) that of the DS, and in any case, in the control group, the *ihDif* was 0.5 Hz between both hemispheres, which can be considered physiological. This normality range has therefore been selected from both the maximum range of the CG and the literature [12,20,21]. However, in the case of DS subjects, the *ihDif* can be higher than the physiological limit of 0.5 Hz, with differences up to 2.5 Hz, clearly indicating a pathological situation. In terms of the difference between the midline channel and parieto-occipital region, the difference for both hemispheres in the control group was similar in the range of 0.5 Hz, the limit of normality. Nevertheless, for the DS group, the difference exceeded the physiological limits, with a maximum ${mlPO}_{left}$ of 1.0 Hz and ${mlPO}_{right}$ of 2.5 Hz.

**Figure 3:**
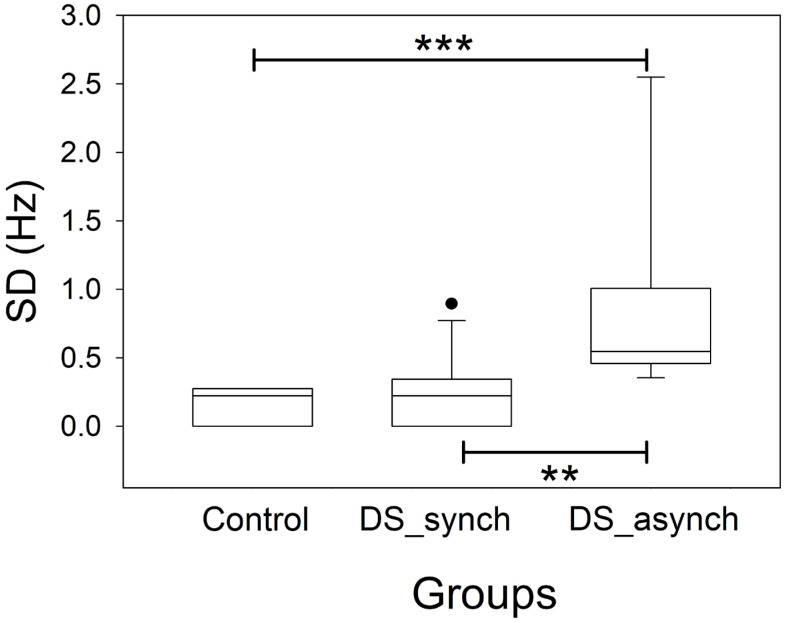
The box plot shows the SDs for the CG (Control, n = 22), DS synchronous (n = 14) and DS asynchronous groups (n = 8). ** *p* = 0.004; *** *p* < 0.001 for Kruskal-Wallis one-way analysis of variance on ranks, Dunn’s post hoc test for pairs.

In the control group, the difference in frequency between hemispheres was $\leq \left|0.5\right|$ Hz, which is considered the limit of physiological behaviour. However, in the DS group, differences greater than this limit were observed in 8/22 participants (36%). Therefore, we can classify the DS group into two subgroups according to pPS behaviour, i.e., asynchronous (differences $>\left|0.5\right|$ Hz) and synchronous. We compared both the subgroups and the control groups (Figure 3).

Therefore, the difference in the pPS in parieto-occipital regions in DS subjects was not physiological in 36% of the subjects, suggesting that alterations in neural networks are involved in posterior alpha band generation. Nevertheless, in this subgroup of DS subjects with asynchrony, the dispersion of all the pPS in the parieto-occipital lobes was greater than that for the CG and synchronous DS subjects.

To assess the possibility that the asynchrony in the posterior region was part of a general alteration in the structure of the bands, we compared this structure in both groups of subjects (Table 3).

The results in this table show that the band structure is similar in both subgroups of subjects with DS. Nevertheless, this non-significance cannot establish similarity when power is inadequate. However, a slight decrease in alpha-band power has been observed in subjects with asynchrony. The significance value is just less than 0.05, so this result should be interpreted with caution.

### 3.3. Bands Structure

The band structure refers to the relationship between the different electroencephalographic bands for a defined lobe. We also analyzed SSE because this is a variable related to the complexity of PS and, therefore, to the differential presence of bands.

**Figure 4:**
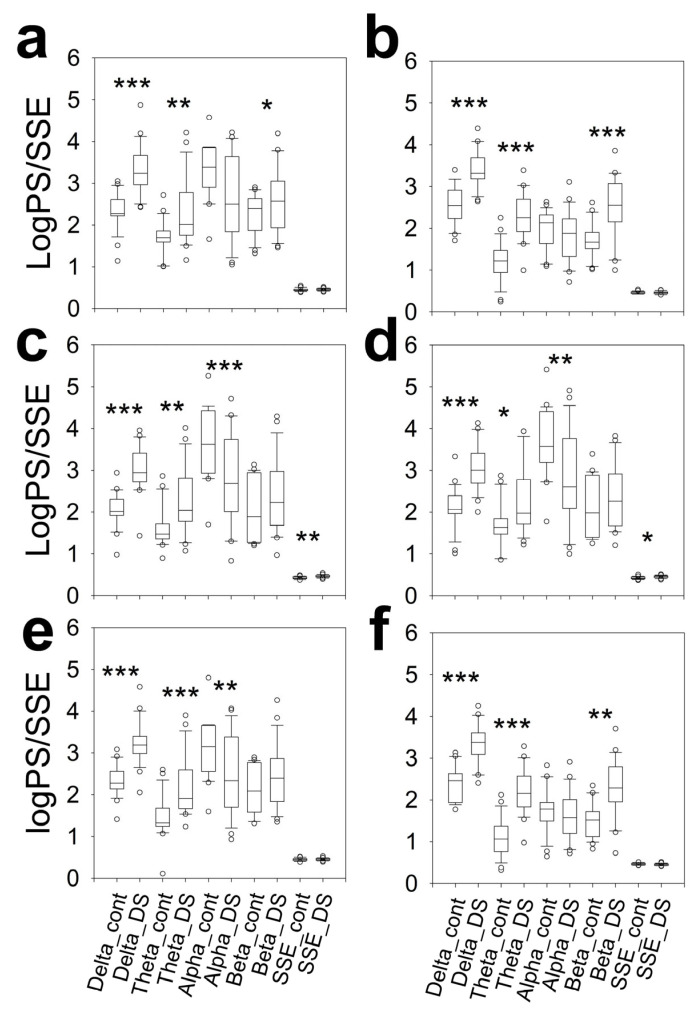
The box plot shows the composition of different bands (in logPS) and the SSE for the control and DS groups. (**a**) Left F, (**b**) right F, (**c**) left PO, (**d**) right PO, (**e**) left T and (**f**) right T regions. Delta_cont = delta band in control; Delta_DS = delta band in DS; Theta_cont = theta band in control; Theta_DS = theta band in DS; Alpha_cont = alpha band in control; Alpha_DS = alpha band in DS; Beta_cont = beta band in control; Beta_DS = beta band in DS; SSE_cont = SSE in control; SSE_Down = SSE in DS; * *p* < 0.05; ** *p* < 0.01; *** *p* < 0.001 for one-way ANOVA on ranks with Bonferroni correction.

Figure 4 shows a generalized increase in the power of the delta and theta bands throughout the scalp in DS subjects. In addition, we observed a highly significant increase in the beta band in the F lobes of the SD patients (Figure 4a,b). The alpha band was lower bilaterally in DS subjects in the PO and T lobes (Figure 4c–f). Finally, SSE was greater in the PO lobes of DS subjects (Figure 4c,d).

Therefore, for 19/30 pairs of variables, there was a highly significant difference between the CG and DS groups, indicating that the band structure is different. The generalized increase in slow bands (delta and theta) is generalized across the scalp, while a decrease in the alpha band is observed in the T and PO lobes. This structure is similar to that described in encephalopathy [20]. However, the significant increase in the beta band at the frontal lobes seems specific to this condition. The complexity of the power spectrum in the PO lobes is greater for DS subjects, considering the highest SSE value.

### 3.4. Scalp Synchronization

**Figure 5:**
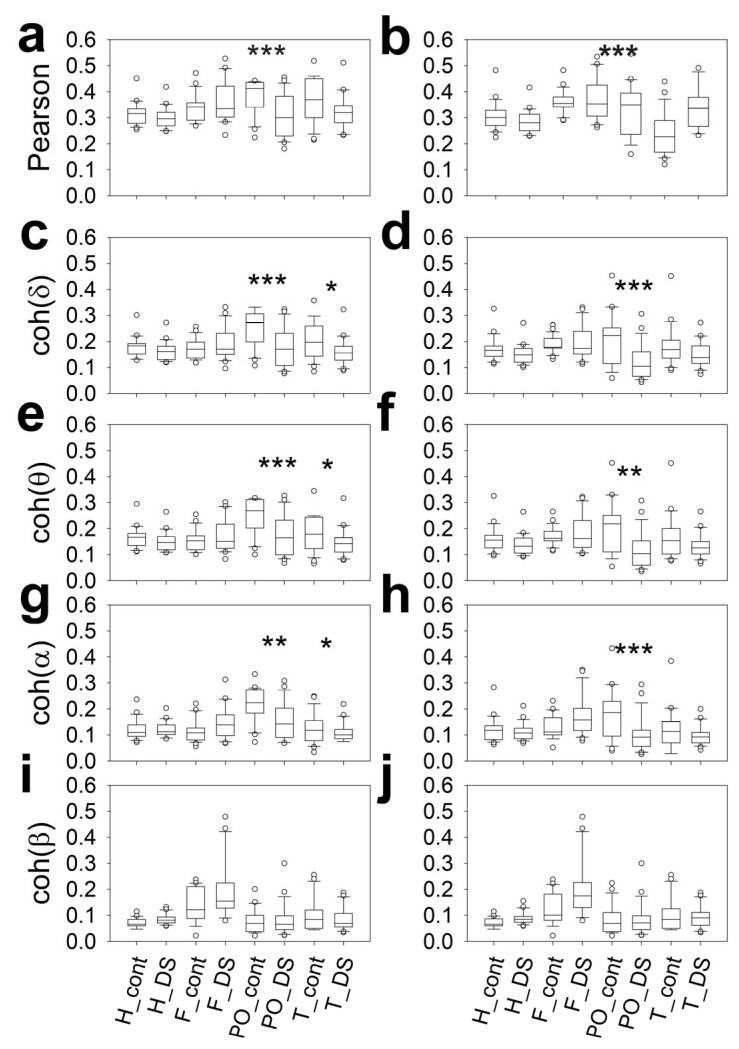
The box plot shows the composition of the synchronization metrics for the control and DS groups. (**a**) Pearson’s correlation coefficient for the left hemisphere, (**b**) Pearson’s correlation coefficient for the right hemisphere, (**c**) coh(δ) for the left hemisphere, (**d**) coh(δ) for the right hemisphere, (**e**) coh(θ) for the left hemisphere, (**f**) coh(θ) for the right hemisphere, (**g**) coh(α) for the left hemisphere, (**h**) coh(α) for the right hemisphere, (**i**) coh(β) for the left hemisphere and (**j**) coh(β) for the right hemisphere. H_cont = hemispheric for control; H_DS = hemispheric for DS; F_cont = frontal in control; F_DS = frontal in DS; PO_cont = parieto-occipital in control; PO_DS = parieto-occipital in DS; T_cont = temporal in control; T_DS = temporal in DS. * *p* < 0.05; ** *p* < 0.01; *** *p* < 0.001 for one-way ANOVA on ranks with Bonferroni correction.

We evaluated the topographical distribution (for hemispheres and lobes) of synchronization by assessing Pearson’s correlation coefficient and coherence. These results can be observed in Figure 5.

Interestingly, both for Pearson correlation and for delta-to-alpha coherence, synchronization in the bilateral PO region was much lower in DS subjects. A decrease in synchronization was also evident in all the bands in the left temporal region of DS subjects. 

It is interesting to observe that no differences in any synchronization metrics between hemispheres was observed for DS subjects.

**Figure 6:**
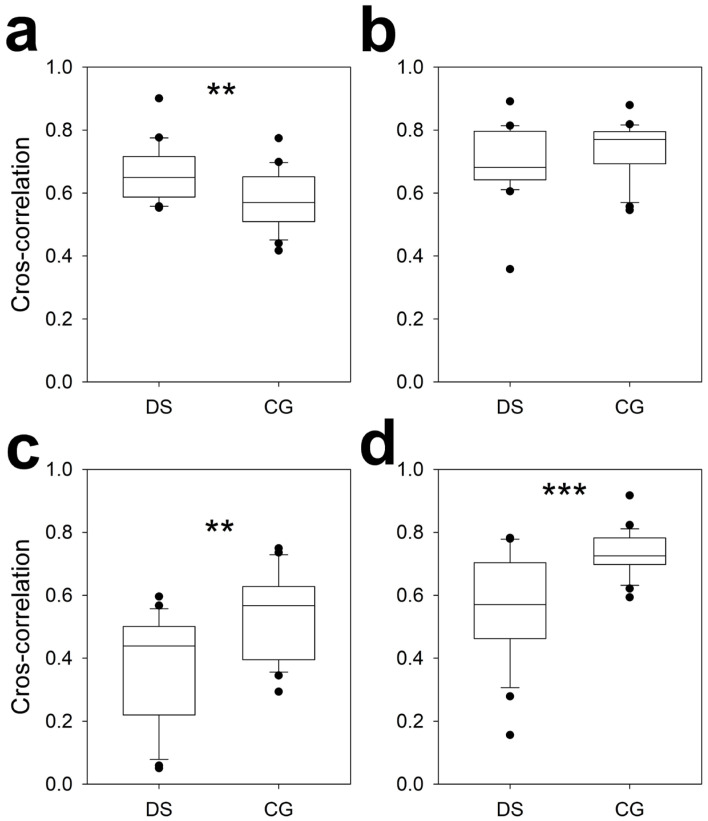
Box plot showing interhemispheric synchronization for both the DS and the CG between paired channels at the (**a**) frontal, (**b**) parietal, (**c**) temporal and (**d**) occipital lobes. ** *p* < 0.01 and *** *p* < 0.001 for two-tailed paired Student’s *t* tests.

The interhemispheric synchronization, addressed by the CC, is shown in Figure 6. The average CC in the F lobes was greater for the DS (Figure 6a) but lower for the T (Figure 6c) and occipital lobes (Figure 6d); however, the average CC did not change for the parietal lobes. In the temporal lobes, the CC of the DS group was 68.5% of the average CC of the CG. The maximum lag values (mean ± SEM [range]) were (for DS/CG respectively), in ms, for the F = −0.44 ± 0.26 [−3.96, 1.95]/0.09 ± 0.16 [−1.95, 1.95], P = −0.98 ± 0.28 [−3.96, 0.0]/−0.98 ± 0.33 [−3.91, 1.93], T = 1.89 ± 0.87 [−10.0, 3.91]/0.09 ± 0.52 [−3.91, 5.86] and O = −0.90 ± 0.71 [−10.0, 9.77]/−0.62 ± 0.39 [−5.86, 1.95]. No differences were observed for the maximum lag at any lobe.

Interestingly, interhemispheric synchronization differed completely between the DS and CG groups, especially in the temporal and occipital lobes. Only the average CC of the parietal lobes was similar between the two groups.

### 3.5. Average Spectra

Although the band composition is evidently related to the average spectra, components in the spectra cannot be directly translated to differences in areas between groups. For example, in a band, there may be an increase in one component and a decrease in another; thus, despite having different components, the total area of that band does not change. Therefore, assessing the average spectra from all the channels is very important.

**Figure 7:**
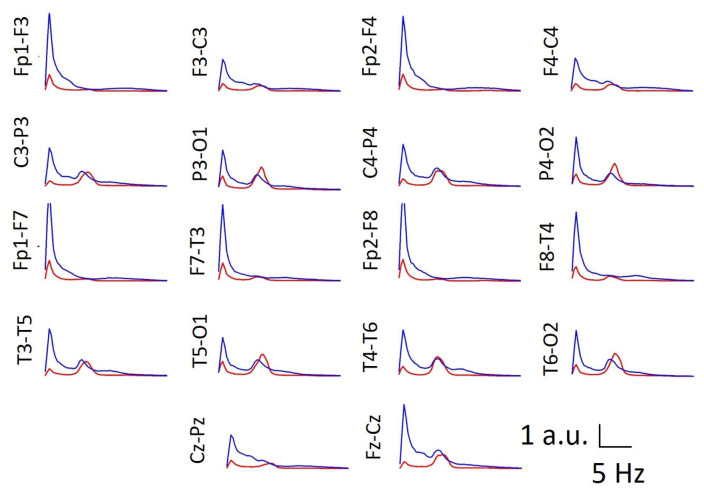
Graph for the average PS of the CG (red) and DS group (blue) for all the channels. All the spectra were normalized to the maximum amplitude of Cz–Pz.

We plotted the average PS for both the control and DS groups for all the channels in Figure 7. There was a generalized increase in the delta and theta bands in the DS group. The high delta value in the frontopolar area can be contaminated with electrooculogram artefacts, but in the remaining channels, the maximum delta peak is of similar magnitude, and consequently, the absence of an antero-posterior gradient precludes contamination from eye movement. Moreover, there is a decrease in the maximum value of the alpha band, along with a general intensification of the beta band, although more prominent in the frontal lobes. The presence of increased beta activity is perfectly clear at the C3–P3/C4–P4 and T3–T5/T4–T6 channels.

### 3.6. Comparison Between Bipolar and AVG Montages

Although a detailed analysis of the similarities and differences between bipolar and AVG montages is beyond the scope of this work, the average spectrum and spatial distribution of EEG bands in scalp have been compared.

**Figure 8:**
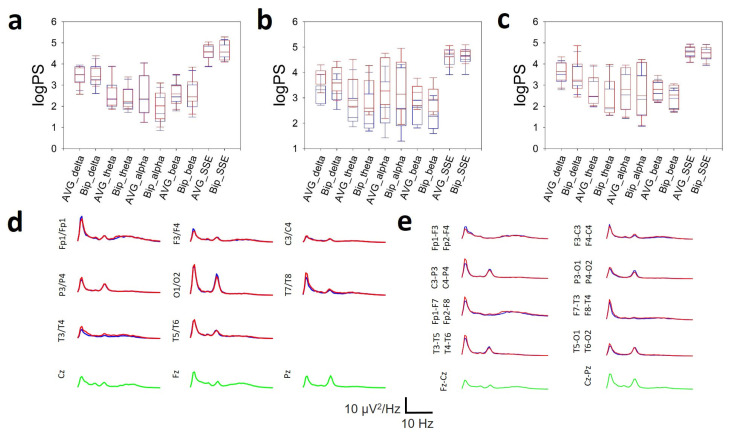
Comparison of average referential (AVG) and bipolar (Bip) montages on the spatial composition of the EEG bands. (**a**) Box plot showing the logPS of the bands and SSE on the frontal, (**b**) parieto-occipital and (**c**) temporal lobes. (**d**) Average spectra for the channels in AVG montage and (**e**) in the bipolar one. Red = right hemisphere, blue = left hemisphere, green = midline.

In Figure 8, we show the distribution by bands at the frontal (Figure 8a), parieto-occipital (Figure 8b) and temporal lobes (Figure 8c). The average spectra per channel for a DS subject are shown in AVG (Figure 8d) and in bipolar montages (Figure 8e).

As can be seen, there is no difference either in the average lobe value of the logPS nor the SSE for any of the EEG bands when comparing both types of montages. This fact is corroborated by the average spectra, which show similar characteristics for both types of montages.

### 3.7. Relationship Between rsEEG Structure and IQ

The relationship between IQ and band structure and synchronization metrics used in the rsEEG of DS individuals was analyzed. Since there is no a priori model that allows the use of a regression function for the data obtained, a non-linear cubic fit by means of least squares was performed.

**Figure 9:**
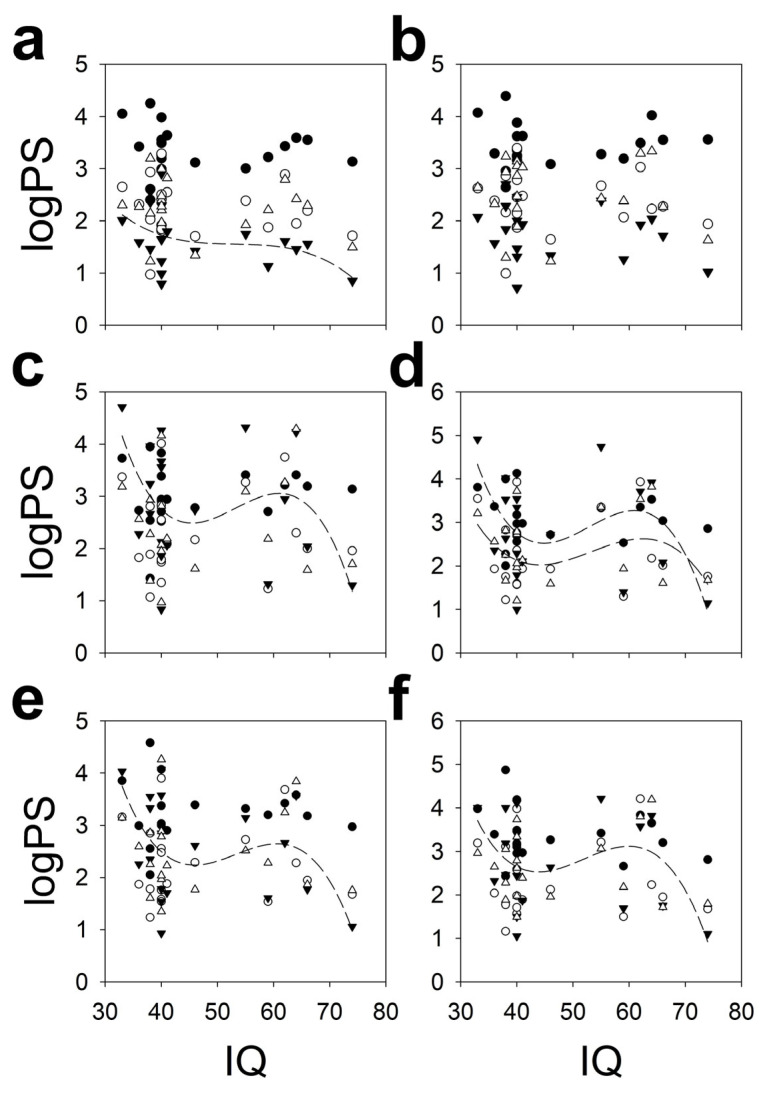
Scalp band structure according to IQ. Only those polynomial functions fitted with r > 0.4224 have been plotted. (**a**) Left frontal lobe, (**b**) right frontal lobe, (**c**) left parieto-occipital lobe, (**d**) right parieto-occipital lobe, (**e**) left temporal lobe and (**f**) right temporal lobe. Delta band = black dots; theta band = empty dots, alpha band = black triangles and medium dash line and beta band = empty triangle and short dash line.

Figure 9 shows the logPS data for each lobe, as a function of IQ. These data have been fitted to a cubic polynomial function. By solving Equation (15) for the conditions of our group, we can find the minimum r to obtain a *p* < 0.05 for a one-tailed Student’s t distribution.

The cubic functions fitted were $\;logF_{alpha}^{left}=11.229-0.5428x+0.0102x^{2}-0.000064x^{3}\;\left(r=0.4340\right),$ $\;log{PO}_{alpha}^{left}=45.48-2.5182x+0.484x^{2}-0.0003x^{3}\;\left(r=0.4642\right),$ $\;logT_{alpha}^{left}=39.384-2.164x+0.0414x^{2}-0.0003x^{3}\;\left(r=0.5002\right),$ $\;log{PO}_{alpha}^{right}=52.776-2.973x+0.0570x^{2}-0.0004x^{3}\;\left(r=0.5249\right)$ $\;and\;logT_{alpha}^{right}=40.1019-2.2670x+0.0448x^{2}-0.0003x^{3}\;\left(r=0.4923\right).$ The correlation between IQ and alpha-band logPS was significant in all lobes, with the exception of the right frontal lobe. In the right parieto-occipital lobe, the correlation between IQ and beta-band was also statistically significant, $log{PO}_{beta}^{right}=33.700-1.8942x+0.0371x^{2}-0.0002x^{3}\;(r=0.4587)$.

**Figure 10:**
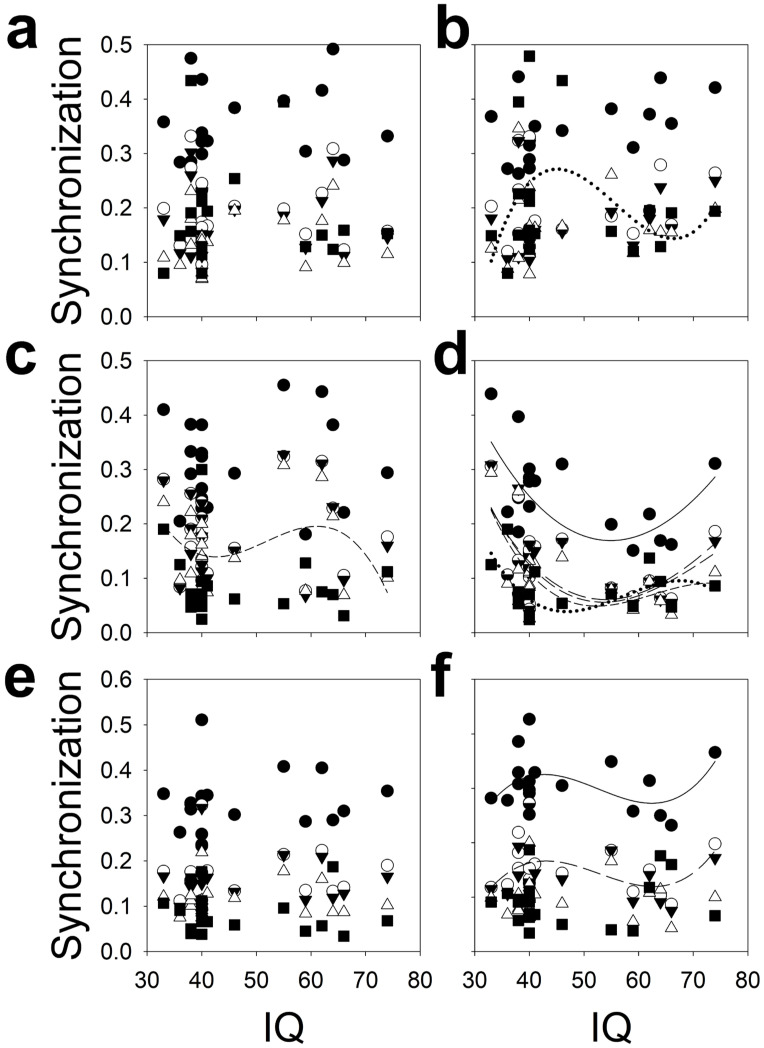
Scalp synchronization structure according to IQ. Only those polynomial functions fitted with r > 0.4224 have been plotted. (**a**) Left frontal lobe, (**b**) right frontal lobe, (**c**) left parieto-occipital lobe, (**d**) right parieto-occipital lobe, (**e**) left temporal lobe and (**f**) right temporal lobe. Pearson’s correlation = black dots and solid line; coh(δ) = empty dots and long dash line, coh(θ) = black triangles and medium dash line, coh(α) = empty triangle and short dash line and coh(β) = black square and dotted line.

We not only analyzed the relationship between IQ and spectral power but we also analyzed its relationship with different synchronization metrics (Figure 10).

In this case, the functions that significantly fit the data are preferentially located in the right hemisphere and, especially, in the parieto-occipital region, where all the regression functions were significant: ${PO}_{Pearson}^{right}=1.4515-0.0510x+0.0006x^{2}-0.00000142x^{3}\;\left(r=0.5416\right),$ $\;{PO}_{coh\left(\delta \right)}^{right}=1.4649-0.061x+0.0008x^{2}-0.00000313x^{3}\;\left(r=0.5871\right),$ $\;{PO}_{coh\left(\theta \right)}^{right}=1.7026-0.0755x+0.0011x^{2}-0.00000489x^{3}\;\left(r=0.5769\right),$ $\;{PO}_{coh\left(\alpha \right)}^{right}=2.2078-0.1086x+0.0018x^{2}-0.0000095x^{3}\;\left(r=0.6121\right)$ $\;and\;{PO}_{coh\left(\beta \right)}^{right}=2.1771-0.1188x+0.0021x^{2}-0.0000125x^{3}\;\left(r=0.5427\right).$ In the case of the right F lobe, only coh(β) was associated with IQ, while for the right T lobe, both Pearson’s correlation and coh(α) were significant $(T_{Pearson}^{right}=-1.5145+0.1115x-0.0022x^{2}+0.0000139x^{3}\;\left(r=0.4587\right)$ $\;and\;T_{coh\left(\delta \right)}^{right}=-1.504+0.1007x-0.0020x^{2}+0.00001247x^{3}\;\left(r=0.4339\right)$). The only regression function statistically significant in the left hemisphere was the coh(α) in the PO lobe, i.e., ${PO}_{coh\left(\alpha \right)}^{left}=2.4012-0.1386x+0.0028x^{2}+0.000017x^{3}\;\left(r=0.4230\right)$.

The different behaviour of the relationship between synchronization and IQ in the right PO lobe and the rest of the scalp is striking. Not only is the correlation coefficient much higher but the morphology of the function is completely different. Therefore, from Figure 10d, the robust relationship between IQ and synchronization at the right PO lobe is evident.

**Figure 11:**
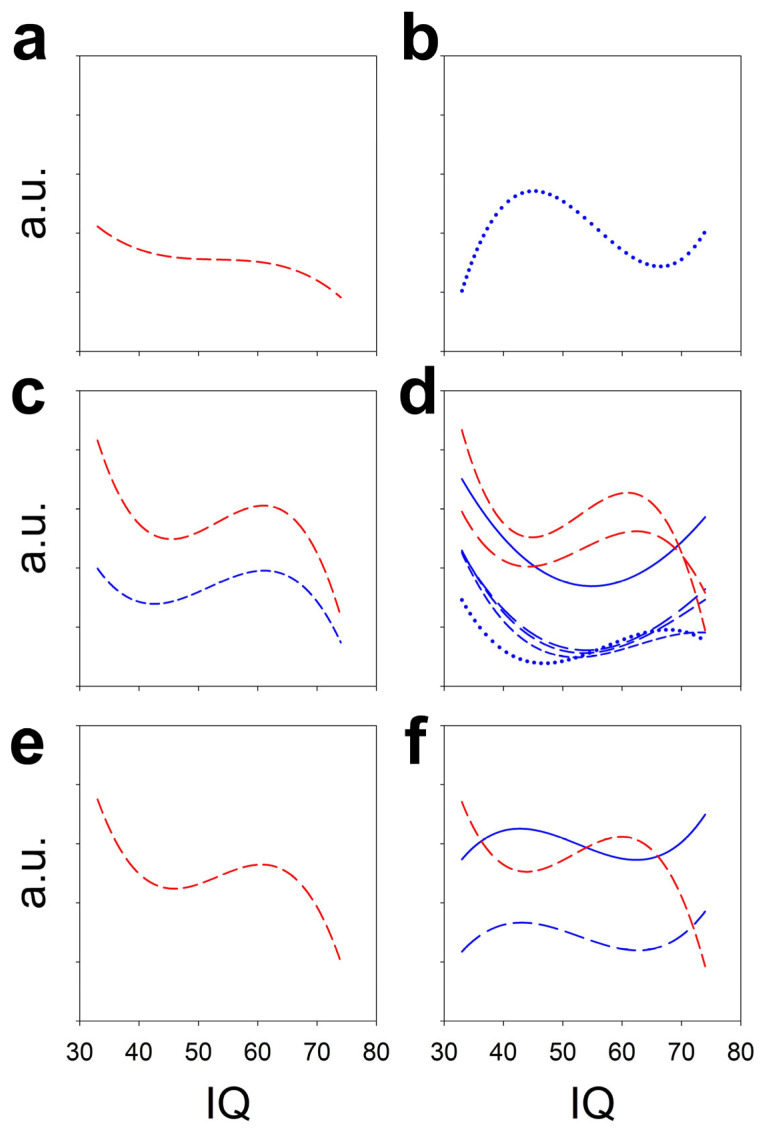
Different polynomial regression functions (only those that are statistically significant) for EEG bands (such as logPS) and timing metrics. (**a**) Left frontal lobe, (**b**) right frontal lobe, (**c**) left parieto-occipital lobe, (**d**) right parieto-occipital lobe, (**e**) left temporal lobe and (**f**) right temporal lobe. Red medium dashed lines = logPS for alpha band, red short dashed lines = logPS for beta band; blue solid line = Pearson’s correlation, blue long dashed line = coh(δ), blue medium dashed lines = coh(θ), blue short dashed lines = coh(α) and blue dotted lines = coh(β).

In Figure 11, we superimposed the lobe-significant polynomial regressions as IQ functions. The logPS functions are shown in red, and the synchronization functions in blue. Since the range of both functions is different, they are shown in arbitrary units to illustrate the different behaviour for power and synchronization measurements across the scalp.

Only at left the PO lobe the regression function for the logarithm of the alpha band and the coh(β) were similar, but for the right PO and T lobes, the regression functions for logPS and synchronization were clearly dissimilar. Therefore, synchronization and PS change in a different way relating to IQ.

The fundamental fact is that the functions of coh(β) in the right F, coh(θ) in the right PO and coh(α) in the left PO are so different that they can be used to identify the IQ based on the synchronization values. Thus, a scalar function can be established such that

$$f(x,y,z):\left[0,1\right]\times \left[0,1\right]\times \left[0,1\right]\to R$$

where x is $F_{coh(\beta )}^{right}$, y is ${PO}_{coh(\theta )}^{right}$ and z corresponds to $F_{coh(\alpha )}^{left}$.

## 4. Discussion

In this study, we described in detail the EEG band structure and topological whole-scalp synchronization of a group of young adults with DS who were age- and sex-matched with euploid control subjects. Furthermore, we analyzed the synchronization properties of the alpha band in PO regions and interhemispheric synchronization. Additionally, we have shown that synchronization in subjects with DS is asymmetric between both hemispheres and specific to different lobes, which allows us to establish a relationship between the scalp synchronization structure and IQ. Unlike most previously published studies, this study assessed the entire scalp and implemented a bipolar montage, which is most commonly used in clinical practice.

Visual analysis of EEG recordings in subjects with DS can be misleading, especially in young subjects. Although recordings may present physiological characteristics, with the presence of an anteroposterior gradient and normal reactivity PDR [12,18,20,21], numerical analysis reveals profoundly abnormal features (see Figure 1). Some older articles have already shown the presence of background and alpha band abnormalities in conventional EEG analysis [22] and during the eye closing/eye opening ratio [23]. In our group, we observed mild to moderate abnormal rsEEG by visual analysis in 52% of the patients. Nevertheless, all the recordings were numerically abnormal, even those that appeared physiologically normal on visual analysis. This is highly relevant from a clinical perspective because, even if the recording does not present epileptiform waveforms and appears physiological, it is highly likely that a numerical analysis will reveal that it is not a normal EEG. Therefore, reporting a recording with these characteristics (e.g., the one shown in Figure 1a) as physiologically normal (despite its similarity to a normal result, as shown in Figure 1d) would constitute a diagnostic error. In addition, there are already clinical trials for the cognitive improvement of DS subjects, so the accurate and early diagnosis of their brain state may be relevant for a personalized treatment and the best way to address the modification in the brain bioelectrical state in response to the treatment. The observed synchronization patterns in different lobes, particularly the right hemisphere, allow for a multivariate relationship with IQ. This previously undescribed finding could enable the objective estimation of subjects’ cognitive status based on rsEEG. Clearly, we are aware that this initial result needs further corroboration and, evidently, it is necessary to increase the number of subjects studied and refine the expression of the functions found, especially by reducing the confidence interval of these functions to ensure the uniqueness of the IQ determination. We are aware that this result needs to be intensively studied before it can be fully established. However, the clear difference in the functions shown in Figure 10 is striking, indicating that the relationship between IQ and EEG structure is a fact of great potential relevance and deserves to be studied in detail.

Indeed, it can be argued that the polynomial fit lacks theoretical justification, but, to our knowledge, no theory exists that predicts the structure of EEG bands as a function of IQ in any group of controls subjects or patients. In fact, the only previous work (except for our own [12]) by John et al. [24] uses fourth-degree polynomial functions. Therefore, obtaining polynomial normative functions is the best that can be achieved in the absence of a theoretical framework. In fact, it is reasonable to expect that obtaining normative functions could serve to delineate theoretical frameworks that currently do not exist. With respect to the scalp band structure, no differences were observed between men and women with DS or between subjects with or without DS [12].

Several anomalies in the band structure in DS subjects have been described. Systematic rsEEG recordings of DS subjects have revealed elevated slow-wave activity, especially in the delta and theta frequency bands, and decreased alpha power; moreover, modifications in the beta band are debated [8].

An association between slower alpha rhythm in posterior regions and cognitive deficits has been described [22,23,25], although other authors have noted the relationship between cognitive impairments and the eyes closed/eyes open ratio [26]. The concept of PDR is not exactly synonymous with the alpha band because the first implies the notion of reactivity, which is not needed to define alpha, which, in fact, refers to oscillations between 8 and 13 Hz [12,20,21]. However, because the PDR usually oscillates into the alpha band, a relationship between both concepts can be established. In accordance with the previous literature, we described a significant decrease in the posterior alpha band at approximately 1 Hz in DS, both in the occipital and parietal regions. Nevertheless, slowing in peak frequency is not the only anomaly observed in DS subjects. In accordance with the chronological evolution of PDR described previously [12], the values of the DS group did not follow those of their age-matched controls but rather followed those of an older cohort. This fact could be interpreted as premature ageing, although there could be other interpretations unrelated to age but related to other brain dysfunctions. Obviously, it will be necessary to analyze other independent ageing biomarkers to determine whether this effect is due to premature ageing or not. Nevertheless, this fact is compatible with the description of an age-dependent decrease in the alpha band in DS subjects [22,27,28]. To further support this hypothesis, the presence of an alpha band in the PO lobes of the DS group was more dispersed and asynchronous than that in the CG, with differences observed between hemispheres higher than 2.5 Hz. We observed a decrease in the mean power of alpha band. We also observed a reduction in alpha band power in subjects with DS and PDR asynchrony. However, this latter finding, in particular, should be studied in a larger number of patients. This fact had not been previously described and probably suggests a derangement of the mechanisms coordinating the alpha generators of both hemispheres.

We observed that not all the DS subjects presented anomalous synchronization for the alpha band (slightly over one-third of the subjects). However, this difference in synchronization is not associated with any other anomalies in either the PS or the synchronization measures. It could be speculated that the anomaly is likely specifically associated with alpha wave generation.

Alpha activity originates from thalamo-cortico-thalamic circuits [29], actively inhibits brain activity, allowing selective attention and supporting executive control processes [8,24,26,29], and is modulated by acetylcholine [30,31,32]. It has been postulated that the reduced release of this neurotransmitter may be the origin of symptomatology in DS individuals [33]. Changes in cholinergic signalling can alter the thalamic alpha rhythm in DS patients, leading to decreases in both alpha power and frequency. In fact, a decrease in the power of the alpha band, not only in the peak frequency, has been extensively described in DS generalized across the scalp but also especially in posterior regions [34,35,36,37,38]. Our results partially overlap with these results because the alpha band in DS subjects did not change compared with that in the CG in frontal regions (2.78 ± 0.21 and 3.3. ± 0.13 for the left lobe and 1.82 ± 0.13 and 1.96 ± 0.10 for the right lobe, respectively, but decreased sharply in the parieto-occipital lobe (3.62 ± 0.17 and 2.78. ± 0.40 for the left lobe and 3.65 ± 0.17 and 2.84 ± 0.24 for the right lobe, respectively, and temporal region (3.15 ± 0.14 and 2.48 ± 0.20 for the left lobe and 1.76 ± 0.12 and 1.61 ± 0.12 for the right lobe, respectively). Therefore, a decrease in alpha band activity in DS was observed in temporal regions and mainly in parieto-occipital regions but not in the frontal lobe.

Delta activity, characterized by slow-wave oscillations below 4 Hz, is regulated by the thalamocortical system, specifically the thalamic reticular nucleus [39], and is strongly influenced by GABAergic inhibitory interneurons [39]. Therefore, the intensified delta activity observed in DS individuals can be directly connected to an increase in the activity of the GABA system, especially when the α4βδ subunits are present in GABA_A_ receptors [29]. Moreover, an increase in the number of inhibitory interneurons and their properties can also contribute to the excess delta band [40,41,42,43,44].

Theta oscillations arise from coordinated neuronal activity in specific brain regions, particularly the hippocampus [45,46], and these waves are associated with several cognitive processes, such as memory, attention and learning, all of which are impaired to different degrees in individuals with DS [47,48].

Delta band increase has been systematically described in DS subjects and is usually associated with a decrease in alpha [26] and mainly in frontal regions [16,34,49,50], increasing in PO regions with age [51] and usually associated with changes in other bands, such as theta or beta [35,37,52].

Our results overlap with those of previous reports, showing a great increase in both delta and theta band power across all the regions of the scalp in individuals with DS. There are likely the most consistent and studied changes in DS subjects.

Beta activity is associated with networks of inhibitory interneurons and usually shows a voltage lower than 20 µV, suggesting that abnormal voltages higher than 25 µV are present [11]. Frontal beta activity is related to metabolic activation, attention, and working memory and increases with mental processes [53,54]. Moreover, benzodiazepines increase beta activity [55], and this band can increase under stress [56]. Notably, an increase in beta activity, especially in regions outside of the frontal regions, may be associated with overexcitation of the cortex and is present in patients with hallucinations, schizophrenia or epilepsy [57,58]. The findings in DS individuals associated with beta activity are the most inconsistent. Some authors have reported a decrease, mainly in parieto-occipital regions [16,34,35,36,37,51], whereas others have described an increase [38,51,58]. In our DS group, we observed a highly significant increase in beta activity in frontal regions (89.0% and 47.1% for the left and right lobes, respectively). Nevertheless, this increase is greater than that shown in Figure 4 since the data in this figure have been calculated from the average values of several channels belonging to the same lobe. We used this method of analysis to maintain consistency of the results with previous work by our group [6,11,12,13,17] and to facilitate a clinically meaningful interpretation. However, the average PS shown in Figure 7 indicates that a prominent beta component is evident at regions posterior to the frontal lobes in parietal areas (C3–P3/C4–P4) and the mid-temporal lobe (T3–T5/T4–T6). In addition to the lobe clustering effect mentioned above, another effect appears to contribute to the lack of statistical significance in other lobes. The average alpha band in DS individuals is slightly shifted towards lower frequencies, as discussed in the PDR analysis. Therefore, although numerically a significant portion of the average activity is included between 8 and 13 Hz (see T3–T5/T4–T6 in Figure 7), i.e., although it would be numerically considered alpha, it actually appears clearly and in an increased form after the proper alpha component; that is, it is functionally equivalent to beta band activity. It shows a marked increase in beta activity in the frontal, anterior parietal, and medial temporal regions. Interestingly, this effect is readily observable through PS analysis but becomes blurred when segmentation by numerical limits derived from recordings of control subjects is used.

It could be argued that normalizing the spectra to the Cz–Pz channel in subjects with DS could systematically bias the visualization of these differences. We believe that this is not actually the case, as can be seen from non-normalized spectra in Figure 1a–c, Figure 8e and Figure A2c, in which the same general characteristics observed in the average spectra of Figure 7 are observed for the individual spectra. No previous papers about the structure of PS in DS have been published. This structure can be addressed according to SSE. We observed that SSE was greater in the DS group in the PO region, whereas in the rest of the scalp, the SSE value was similar for both groups. Therefore, the PS complexity was greater in DS subjects in those regions. Compared with those in control subjects, PS channels are indeed more complex in DS subjects, as shown in Figure 7.

Another aspect that has not been precisely studied in subjects with DS is synchronization. It has been shown that the intrahemispheric coherence in the rsEEG was higher in the DS group than in the controls. The age-specific development of coherence in the interhemispheric parieto-occipital region was almost identical in children with Down syndrome and controls, both with open eyes and closed eyes [59]. However, coherence deficiencies in the DS group became more prominent with increasing age from school age onwards [15]. In addition, the directed transfer function showed a globally prevailing information transfer from the right to the left occipital areas in young control subjects and in the opposite direction in DS patients [16]. On the other hand, the global organization of the DS brain does not resemble a small-world network but rather a random network [14].

Our results revealed a significant decrease in Pearson’s correlation in both PO lobes of individuals with DS. Coh is the frequency domain equivalent to the time-domain cross-correlation function [13,60], and it can be made equivalent to the total coherence. Band analysis revealed significant decreases in coh(δ), coh(θ), and coh(α) in the PO regions of DS subjects but a greater difference with respect to the CG in the right hemisphere. However, in the right hemisphere, no differences were observed in the coh of these bands, while in the left hemisphere, the values of these metrics in the DS group were also lower than those in the control group. With respect to coh(β), we observed that in the DS group, the average hemispheric value increased, along with an increase in this metric in the right frontal lobe and a decrease in the left temporal region.

Although it is not a strictly directional measure, the CC can be used to estimate the direction of interaction between two signals. Even though it does not allow identification of the direction of transfer, as in the directed transfer function or Granger causality, the maximum lag allows us to suspect directionality and its magnitude. We addressed interhemispheric synchronization by comparing the means of paired channels in the F, T, P and O lobes. We observed that the CC was greater in the F lobe, the CC was similar in the P lobe, and the CC was much lower in the T and O lobes in the DS group than in the control group. The maximum lag was near zero for all the pairs of channels. Therefore, we did not find the directionality described by other authors [16]. However, it should be noted that neither the metrics used nor the montage used for analysis are the same, so these results are not surprising. On the other hand, although CC has a temporal dimension, it is a method directly related to Pearson’s correlation [13], so it is not surprising to find similar results from both metrics. On the other hand, the near-zero maximum lag value could be attributed to a volume conduction effect. However, it should be noted that bipolar montage is less susceptible to volume conduction effects than AVG [13].

Our results partially overlap with those previously published, such as the well-established increase in delta and theta activity and the decrease in alpha power in occipital regions. Nevertheless, we found results that were not previously described, such as posterior alpha band instability, which probably has a local mechanism, since it is not associated with other alterations. Similarly, in contrast to other previous studies, we showed a marked increase in frontal and central beta activity, and we described in detail topographical differences in average synchronization, mainly in the PO lobes, and an inhomogeneity in T coherence.

Some of these differences can be explained by methodological differences. In numerous articles, especially older ones, it was not common practice to use all the electrodes of the 10–20 system but rather a selected number of them. Furthermore, in all the reviewed articles, both the spectral and synchronization measurements were obtained from reference montages, either on average, to the Cz electrode or to the mastoid electrodes. Unfortunately, there are no studies comparing PS or synchronization results in average and bipolar montages in recordings on the same group of subjects. Although a comprehensive study of the topographic band distribution and the different synchronization metrics would require an entire article, which obviously exceeds the scope of this work, we have verified in a subgroup of subjects with DS that the use of bipolar montages does not modify the band structure with respect to the AVG reference montage. This finding, which has not been previously reported, indicates that the results of our study are directly comparable with those in the literature. Bipolar montages have advantages and disadvantages, as do average montages, but the voltage time series obtained from them are clearly not artefacts (in the sense of signals of non-cerebral origin), although both of them, bipolar and average, have different amplitude, phase, and, to some extent, spectral composition characteristics. Numerical analysis of different time series will also yield different results, but the nature of these differences is not yet fully understood [12,13,61,62,63].

There are some limitations to our study that should be addressed in the future. First, the number of patients was limited. This implies that the statistical power of most of our tests was less than 0.8. The low value of statistical power (the probability of avoiding a type II error) occurs when the sample size is too small and means that we must interpret negative findings cautiously. Therefore, we must be aware that it is possible that we incorrectly accepted the null hypothesis. Obviously, this option is more conservative than accepting alternative hypotheses without adequate statistical support. On the other hand, increasing the number of patients will ultimately rise the statistical power and reduce this type of error. Furthermore, the difference in methods can lead to inconsistent results, although we have verified that, at least with regard to the average PS values in scalp, there are no differences between bipolar and AVG montages. However, the preferred use of bipolar montage in clinical practice suggests that, at least in this field of application, the values derived from this type of montage are highly relevant.

## 5. Conclusions

The protocol for routine rsEEG recorded with a double-banana bipolar montage in subjects with DS has been described for the assessment of band structure, synchronization, PDR and, maybe, IQ. Therefore, this work highlights the feasibility of a method that is easy for clinicians to use in daily routine practice. In this sense, it is important to be aware that visually evaluated, even apparently normal, rsEEG can present significant deviations from physiology in numerical analysis. Thus, qEEG should be implemented in the routine analysis of DS subjects.

Subjects with DS showed a significant increase in the power of the delta and theta bands across the entire scalp, along with a decrease in the power of the alpha band in the posterior half of the scalp. This alpha activity is not only diminished but also exhibits characteristics corresponding to older euploid subjects and is asynchronous between both hemispheres in one-third of the individuals.

In our group of young adult subjects with DS, the beta band was significantly increased in the frontal lobes and adjacent regions, such as the parietal and medial temporal regions. In contrast to other authors, we did not observe a decrease in the strength of this band in any scalp region.

We characterized a generalized decrease in PO synchronization, which showed different alterations. Notably, left temporal synchronization is also lower in this group of subjects than in the controls. Although no changes in directionality were detected, differences between controls and DS patients were observed in terms of the synchronization of specific channel pairs; notably, synchronization was greater in subjects with DS in the frontal lobe and much lower in the occipital and temporal regions.

Finally, a marked interhemispheric asynchrony has been observed, especially in the parieto-occipital region. This anomaly is apparently related to IQ and therefore could serve as an estimate of cognitive abilities in these subjects.

## Figures and Tables

**Table 1:** Comparison between sexes in the DS group. Two-tailed Student’s *t* tests were used to assess significance.

Topography	Men (n = 14)	Women (n = 8)	*p*
Left hemisphere			
Frontal			
Delta	3.237 ± 0.124	3.543 ± 0.154	0.144
Theta	2.115 ± 0.166	2.374 ± 0.137	0.300
Alpha	1.570 ± 0.168	1.706 ± 0.171	0.577
Beta	2.244 ± 0.207	2.337 ± 0.183	0.740
SSE	0.4559 ± 0.0797	0.4551 ± 0.0624	0.939
Parieto-occipital			
Delta	2.849 ± 0.146	3.337 ± 0.140	0.039 *
Theta	2.118 ± 0.232	2.555 ± 0.211	0.223
Alpha	2.488 ± 0.241	3.303 ± 0.443	0.134
Beta	2.212 ± 0.226	2.739 ± 0.293	0.174
SSE	0.4608 ± 0.0976	0.4562 ± 0.0104	0.753
Temporal			
Delta	3.122 ± 0.123	3.446 ± 0.199	0.159
Theta	2.126 ± 0.214	2.364 ± 0.188	0.172
Alpha	2.321 ± 0.233	2.770 ± 0.361	0.315
Beta	2.348 ± 0.212	2.622 ± 0.234	0.398
SSE	0.4590 ± 0.0882	0.4568 ± 0.0971	0.868
Right hemisphere			
Frontal			
Delta	3.293 ± 0.109	3.697 ± 0.147	0.038 *
Theta	2.196 ± 0.168	2.495 ± 0.108	0.225
Alpha	1.734 ± 0.170	1.965 ± 0.179	0.361
Beta	2.348 ± 0.212	2.622 ± 0.234	0.384
SSE	0.4617 ± 0.0936	0.4624 ± 0.0994	0.963
Parieto-occipital			
Delta	2.932 ± 0.156	3.381 ± 0.138	0.068
Theta	2.114 ± 0.228	2.536 ± 0.242	0.101
Alpha	2.570 ± 0.241	3.318 ± 0.473	0.187
Beta	2.228 ± 0.201	2.702 ± 0.259	0.169
SSE	0.4547 ± 0.0100	0.4527 ± 0.0259	0.885
Temporal			
Delta	3.211 ± 0.134	3.533 ± 0.231	0.209
Theta	2.210 ± 0.238	2.491 ± 0.219	0.219
Alpha	2.465 ± 0.231	3.046 ± 0.411	0.242
Beta	2.428 ± 0.208	2.838 ± 0.243	0.219
SSE	0.4564 ± 0.0176	0.4617 ± 0.0113	0.701

* *p* < 0.05.

**Table 2:** Comparison of PDR properties between control and DS subjects.

Variable	Control		DS	
	Absolute Frequency	Range	Absolute Frequency	Range
*ihDif*	3	−0.5, 0.5	8	−2.5, 1.5
${mlPO}_{left}$	10	−0.5, 0.5	10	−1.0, 1.0
${mlPO}_{right}$	13	−0.5, 0.5	10	−2.5, 1.5

**Table 3:** Comparison between the synchronous and asynchronous alpha bands in DS subjects. Two-tailed Student’s *t* tests were used to assess the significance.

Lobe	Synchronous (n = 14)	Asynchronous (n = 8)	*p*
Left hemisphere			
Frontal			
Delta	3.401 ± 0.138	3.254 ± 0.137	0.492
Theta	2.235 ± 0.149	2.164 ± 0.204	0.778
Alpha	1.802 ± 0.161	1.301 ± 0.125	0.0449 *
Beta	2.333 ± 0.172	2.181 ± 0.275	0.626
SSE	0.4548± 0.0592	0.45751 ± 0.0224	0.841
Parieto-occipital			
Delta	3.079 ± 0.175	2.935 ± 0.0941	0.563
Theta	2.351 ± 0.222	2.148 ± 0.269	0.576
Alpha	3.112 ± 0.321	2.210 ± 0.193	0.060
Beta	2.515 ± 0.263	2.209 ± 0.210	0.436
SSE	0.4546 ± 0.102	0.4671 ± 0.0609	0.352
Temporal			
Delta	3.291 ± 0.167	3.151 ± 0.0803	0.554
Theta	2.272 ± 0.193	2.110 ± 0.255	0.413
Alpha	2.772 ± 0.273	1.982 ± 0.163	0.022 *
Beta	2.538 ± 0.221	2.289 ± 0.210	0.424
SSE	0.4551 ± 0.0765	0.4636 ± 0.0423	0.470
Right hemisphere			
Frontal			
Delta	3.503 ± 0.125	3.329 ± 0.147	0.380
Theta	2.344 ± 0.149	2.236 ± 0.197	0.667
Alpha	2.007 ± 0.168	1.488 ± 0.122	0.044 *
Beta	2.587 ± 0.186	2.391 ± 0.307	0.901
SSE	0.513 ± 0.0993	0.4631 ± 0.07592	0.963
Parieto-occipital			
Delta	3.169 ± 0.162	2.967 ± 0.168	0.400
Theta	2.359 ± 0.216	2.108 ± 0.293	0.453
Alpha	3.131 ± 0.326	2.335 ± 0.246	0.388
Beta	2.510 ± 0.220	2.208 ± 0.233	0.169
SSE	0.4517 ± 0.0952	0.4581 ± 0.0127	0.682
Temporal			
Delta	3.389 ± 0.175	3.223 ± 0.139	0.523
Theta	2.367 ± 0.207	2.216 ± 0.312	0.539
Alpha	2.928 ± 0.291	2.235 ± 0.233	0.119
Beta	2.648 ± 0.206	2.454 ± 0.272	0.576
SSE	0.4567 ± 0.0568	0.512 ± 0.0791	0.601

* *p* < 0.05.

## Data Availability

The data presented in this study are available on request from the corresponding author due to ethical reason.

## Abbreviations

The following abbreviations are used in this manuscript:

alpha_o_	Average alpha band at occipital lobe
alpha_p_	Average alpha band at parietal lobe
CC	Cross-correlation
CG	Control Group
coh	Coherence
DS	Down syndrome
F	Frontal lobe
FFT	Fast Fourier Transform
H	Hemisphere
ihDif	Interhemispheric difference
IQ	Intelligence quotient
logPS	Logarithm transform of PS
${mlPO}_{left}$	Difference between the Cz–Pz channel and P3–O1
${mlPO}_{right}$	Difference between the Cz–Pz channel and P4–O2
O	Occipital lobe
PDR	Posterior Dominant Rhythm of the DS/CG
pPS	Peak Frequency of power spectrum at alpha band
P	Parietal lobe
PO	Parieto-occipital lobe
PS	Power Spectrum
rsEEG	Resting state EEG
SD	Standard deviation for the parieto-occipital channels
SSE	Shannon’s spectral entropy
T	Temporal lobe

## Appendix A

**Table A1:** Visual features of EEG for different diagnosis.

EEG Diagnosis	Gradient	PDR	Delta/Theta Power
Physiological	Yes	Alpha	-
Mild slowing	Yes	<8 Hz	-
	-	<8 Hz	Slight increase
Encephalopathy	-	Clearly slow or absent	Clearly increase

**Table A2:** Comparison between subjects with DS and individuals with DS and treated with levothyroxine or with DS and diagnosed with sleep apnoea. One-way ANOVA with Bonferroni correction.

	Mean	SEM	Mean	SEM	Mean	SEM	*p*
**Delta**							
Left F	3.341	0.138	3.313	0.17	3.696	0.305	0.639
Left PO	3.019	0.173	3.034	0.162	3.279	0.336	0.786
Left T	3.26	0.139	3.181	0.147	3.495	0.543	0.885
Right F	3.453	0.135	3.373	0.134	3.749	0.34	0.455
Right PO	3.132	0.168	3.074	0.198	3.223	0.397	0.387
Right T	3.389	0.135	3.194	0.193	3.598	0.636	0.757
**Theta**							
Left F	2.185	0.186	2.223	0.0962	2.428	0.331	0.668
Left PO	2.424	0.247	2.182	0.308	2.225	0.304	0.489
Left T	2.362	0.228	2.118	0.223	2.103	0.381	0.501
Right F	2.287	0.188	2.339	0.0879	2.408	0.282	0.328
Right PO	2.413	0.25	2.245	0.324	2.118	0.367	0.757
Right T	2.453	0.249	2.228	0.259	2.191	0.47	0.651
**Alpha**							
Left F	1.581	0.185	1.644	0.118	1.859	0.385	0.151
Left PO	2.941	0.325	2.834	0.465	2.627	0.669	0.851
Left T	2.648	0.294	2.496	0.316	2.264	0.645	0.754
Right F	1.775	0.186	1.864	0.147	1.984	0.401	0.465
Right PO	2.991	0.32	2.948	0.508	2.638	0.697	0.354
Right T	2.809	0.283	2.713	0.381	2.455	0.78	0.775
**Beta**							
Left F	2.104	0.196	2.405	0.222	2.74	0.288	0.658
Left PO	2.438	0.297	2.453	0.209	2.398	0.272	0.595
Left T	2.508	0.262	2.475	0.151	2.352	0.265	0.687
Right F	2.358	0.233	2.69	0.212	2.833	0.303	0.754
Right PO	2.444	0.254	2.476	0.24	2.304	0.261	0.486
Right T	2.656	0.254	2.529	0.185	2.473	0.312	0.685
**SSE**							
Left F	0.454	0.0631	0.684	0.0111	0.462	0.0764	0.456
Left PO	0.504	0.105	0.667	0.0116	0.467	0.0666	0.457
Left T	0.532	0.0956	0.661	0.0856	0.4515	0.0186	0.558
Right F	0.504	0.0825	0.594	0.0128	0.464	0.0101	0.621
Right PO	0.469	0.0107	0.508	0.0107	0.4653	0.0481	0.471
Right T	0.532	0.090	0.536	0.0109	0.4553	0.0163	0.665
